# Assessing the Total Factor Productivity of Cotton Production in Egypt

**DOI:** 10.1371/journal.pone.0116085

**Published:** 2015-01-27

**Authors:** Xosé A. Rodríguez, Yahia H. Elasraag

**Affiliations:** 1 Department of Quantitative Economics, University of Santiago de Compostela, Santiago de Compostela, Spain; 2 Department of Agricultural Economics, Cairo University, Gammaa St., Giza, 12613, Egypt; Universidad Veracruzana, MEXICO

## Abstract

The main objective of this paper is to decompose the productivity growth of Egyptian cotton production. We employ the stochastic frontier approach and decompose the changes in total factor productivity (CTFP) growth into four components: technical progress (TP), changes in scale component (CSC), changes in allocative efficiency (CAE), and changes in technical efficiency (CTE). Considering a situation of scarce statistical information, we propose four alternative empirical models, with the purpose of looking for convergence in the results. The results provide evidence that in this production system total productivity does not increase, which is mainly due to the negative average contributions of CAE and TP. Policy implications are offered in light of the results.

## Introduction

The agricultural sector is considered as one of the major sectors in Egypt’s economy, where its contribution to the country’s GDP is currently around 14% [1]. The Egyptian agriculture has at least six features that make it unique among other agricultural systems [2]. First, the limitedness of the arable lands, given Egypt’s location in a dry desert region. Second, the dependence on the Nile water for irrigation; in fact the agricultural sector is the main consumer of the Nile water, accounting for around 84% of its consumption. Third, the intensive cropping systems, which are based on the scarcity of land resources and the availability of irrigation water supply. Fourth, the predominance of the small holding pattern. Fifth, an intensive use of inputs, particularly chemical fertilizers and labor, and finally a cropping pattern based on many basic groups, such as cereals, legumes, fibers, sugar crops, oil crops, fodder, fruits, vegetables and horticultural crops.

The production of cotton has great importance in the Egyptian agricultural sector. First, because cotton production is a very intensive activity in the use of labor and it is carried out principally by small family exploitations. Additionally, it generates new opportunities of employment in the subsequent processes (e.g. ginning, transportation, commercialization and the local textile industry). Second, cotton production is important in Egypt because it helps to guarantee the food security for population [1]. This contribution to the food security takes several forms. To begin with, cotton production is a source of income for rural families, who can then afford the basic products (milk, fruits, vegetables, clothes, etc). Also the exports of cotton enable the import of food. In addition, the oil extracted from the cotton seeds is used for culinary purposes, and the chaff (oilcake) is used to feed animals, since it is rich in proteins. Finally, the production of cotton is important as a raw material to impel the textile industry in Egypt.

Considering the importance of cotton production in Egypt, two issues are surprising. First, there are not empirical studies on this sector that analyze the evolution of total factor productivity (TFP) from its determinants. Second, a substantial decline was observed in cotton production (period 2004–2008), while the production of other agricultural products, such as sugarbeet, fruits, vegetables, rice and wheat, increased in Egypt in a remarkable way [1]. In addition, the decline of cotton production in Egypt took place in a context in which the Egyptian economy was growing and so was the Egyptian cotton textile industry, while the production, consumption, exports, and imports of cotton did not decrease at the global level but remained stable [3].

Some studies on cotton in Egypt, such as [4], enumerate multiple causes (at the national and international level) that can explain the evolution of cotton production. One of the suggested causes is the low productivity growth, given that productivity is an important determinant of the evolution of product competitiveness. And competitiveness is one of the principal factors that determine increases in the demand of products and, as a consequence, increases in the possibilities of production.

In view of the above considerations, the main objective of this study is to carry out a disaggregate analysis (by provinces) of TFP in Egyptian cotton production. In addition, it aims to analyze the evolution of TFP from its determinants (including the efficiency). On the basis of these results, this paper aims to provide evidence on the possible problems affecting the cotton production system in Egypt. One of the peculiarities of this study is the use of scarce data. To overcome this limitation, we propose four alternative empirical models to compare the results obtained. If the results obtained by the different models are similar, they can be considered to be more consistent and meaningful.

To meet our objectives the rest of the paper is organized as follows. In the subsequent section we present a brief review of our methodology. Section three deals with the relationship between the statistical information available and the empirical models proposed. Section four presents the results. The final section presents the main conclusions and policy implications.

## Methodology

Solow [5] proves that, under constant returns to scale, in a long run competitive equilibrium technical progress may be the only source of total productivity growth. Following this proposal, many studies of productivity growth in agriculture tended to compute productivity as a residual after accounting for input growth; i.e., productivity growth was identified with technical progress. Currently, some authors continue to analyze productivity growth in agriculture computing productivity as a residual, using index numbers such as Törnqvist (e. g., [6, 7, 8, 9, 10]). In these studies firms are assumed to maximize profits, and thus they are assumed to be efficient. In reality, however, even if firms have as an objective to obtain the maximum benefit, not all of them are able to achieve it, and as a consequence inefficiencies may arise. If there is inefficiency, it is necessary to take into account its effect over productivity growth. It is likewise crucial to consider the impact on productivity of non-constant returns to scale and of the violation of the various conditions necessary for long run competitive equilibrium.

There exist two main types of approaches that can be applied to estimate efficiency and decomposing productivity growth: data envelopment analysis (DEA) and stochastic frontier analysis (SFA) [11]. In general, the SFA approach has been preferred in the agricultural economics literature [12, 13, 14]. The most important potential advantage of SFA is that it can separate noise in data from variations in efficiency. Given the inherent variability of agricultural production, the assumption that all deviations from the frontier are associated with inefficiency (as assumed in DEA approach) is difficult to accept in this sector.

Following previous literature in the agricultural field (e. g., [13, 15, 16, 17, 18, 19, 20]), the starting point for a measure of productivity can be a stochastic frontier production function:

$$Q_{it}=f(X_{it},t\text{;}\alpha )e^{v_{it}-u_{it}}$$ (1)

where *Q_it_* is the production of the *i-th* firm (*i* = 1, 2 … *N*) in the *t-th* time period (*t* = 1, 2 … *T*); *f*(·) is the production technology; *X_it_* is a vector of J input quantities of the *i-th* firm in the *t-th* time period; t is the time trend variable; *α* is a vector of unknown parameters to be estimated; *v_it_* is a vector of random variables which are assumed to be iid. $N(0,\sigma_{v}^{2})$, and independent of the *u_it_*, which is a vector of non-negative random variables assumed to account for technical inefficiency in production. There are several specifications that make the technical inefficiency term *u_it_* time-varying. In this paper we use the Error Components Model (ECM) specification proposed by [21], and the Technical Efficiency Effects Model (TEEM) specification proposed by [22].

In ECM, the inefficiency term *u_it_* is defined as:

$$u_{it}=u_{i}\exp(-\eta [t-T])$$ (2)

where the distribution of *u_i_* is taken to be the non-negative truncation of the normal distribution$N(\mu ,\sigma_{u}^{2})$, and *η* is a parameter that represents the rate of change in technical inefficiency. The positive (negative) value of *η* is associated with improvements (deterioration) in cotton production technical efficiency over time.

In TEEM, the non-negative random variables (*u_it_*) are assumed to be independently distributed as truncations at zero of the $N(m_{it},\sigma_{u}^{2})$ distribution; where:

$$m_{it}=z_{it}\delta$$ (3)

where *z_it_* is a vector of variables which may influence the efficiency of a firm and *δ* is a vector of parameters to be estimated.

Differentiating the production technology in Eq. (1) with respect to time and rearranging terms we have that:

$$\frac{d\ln f(.)}{dt}=TP+\sum_{j}^{}\varepsilon_{j}\overset{}{{\dot{x}}_{j}}$$ (4)

where *TP* = ∂lnƒ(.)/∂t represents the technical progress or technical change; *ε_j_ = ∂*lnƒ(.)/∂ln*x*_j_ is the elasticity of output with respect to the *j*th input, and a dot over *x* indicates its rate of change.

Differentiating the log of Q in Eq. (1) with respect to time, the change in production can be represented as:

$$\overset{}{\dot{Q}}=TP+\sum_{j}^{}\varepsilon_{j}\overset{}{{\dot{x}}_{j}}-\frac{du}{dt}$$ (5)

In Eq. (3), the overall productivity change is affected not only by TP and changes in input use, but also by the change in technical efficiency (−*du*/*dt);* thus if *du / dt* is positive (negative), technical efficiency deteriorates (improves) over time.

To examine the effects of technical progress and changes in efficiency on TFP growth, the traditional definition for productivity total growth is used; that is, TFP is taken as the residual resulting from the output growth unexplained by input growth:

$$T\text{​}\overset{}{\text{​}\dot{F}}\text{​}P=\overset{}{\dot{Q}}-\sum_{j}^{}s_{j}\overset{}{{\dot{x}}_{j}}$$ (6)

where *S_j_* is the share of input *j* in production costs.

By substituting Eq. (5) in Eq. (6), Eq. (6) is rewritten as:

$$T\text{​}\overset{}{\dot{F}\text{​}}\text{​}P=TP-\frac{du}{dt}+(RS\;\text{​}-1)\sum_{j}^{}\lambda_{j}\overset{}{{\dot{x}}_{j}}+\sum_{j}^{}(\lambda_{j}-S_{j}){\overset{}{\dot{x}}}_{j}$$ (7)

where $RS=\sum_{j}\varepsilon_{j}$ denotes the measurement of returns to scale and *λ_j_* = *ε_j_*/*RS*.

In Eq. (7) the total factor productivity growth is decomposed into four components: technical progress (TP), changes in technical efficiency (CTE = -*du*/*dt*), changes in the scale component [CSC = (RS-1)Σ*λ_j_ẋ_j_*] and changes in allocative efficiency [$CAE=\sum_{j}(\lambda_{j}-S_{j}){\overset{\overset{}{}}{\dot{x}}}_{j}$].

The interpretation of the four components in equation (7) is the usual. TP measures the change in frontier output: TP is positive (negative) if exogenous technical change shifts the production frontier upward (downward) for a given level of inputs. *CTE* can be interpreted as the rate at which an inefficient firm catches up to the production frontier. Depending on whether *RS*> 1, *RS* < 1 or *RS* = 1, positive scale effects, negative effects or non-scale effects, respectively, will exist. Finally, *CAE* measures inefficiency in resource allocation resulting from deviations of input prices from their marginal value.

### Data and empirical models

In this study we use panel data at the province level covering the period 1990–2008. The dataset was obtained from “Agricultural Statistics”, a publication by the Ministry of Agriculture and Land Reclamation [23] which contains information on the main nine cotton-producing provinces. One of the limitations of this publication is that it offers too scarce data for a detailed study of total productivity.

The most important problem from the empirical point of view is that, in this publication, the only real and direct data provided are the production (Q) and the cotton area (X_A_). The other inputs—labor input (X_L_), capital input (X_K_) and materials input (X_M_)—can be calculated according to the input rate per hectare for every year (also published by the same source):

$$\begin{array}{l} X_{Lit}=l_{t}*X_{Ait}\\ X_{Kit}=k_{t}*X_{Ait}\\ X_{Mit}=m_{t}*X_{Ait}\; \end{array}$$ (8)

where *l_t_, k_t_ and m_t_* are the input rates per hectare for labor, capital and materials, respectively, in year *t* (t = 1, 2, …T). This method of estimation of the quantities of the productive factors (labor, capital and materials) may increase the degree of correlation between them. This might be problematic when considering the four inputs jointly in a production function.

Additionally, there is a lack of province-level statistical information on specific variables such as education, credit use, infrastructures, land quality, average size of plantations, composition of labor or characteristics of machinery, which may have a significant impact on the behavior of efficiency and productivity. There is also no data at the farm level, such as age, farm income, off-farm income, womens’ participation, experience or farm size. Table 1 presents the summary statistics for the variables used in the analysis for the total sample (171 observations). All of these variables show high variability across the sample, since all of them have a very high standard deviation in relation to its average (the Pearson’s coefficient of variation is found to be superior to 62% for all of them).

**Table 1:** Summary statistics.

**Variables**	**Units**	**Minimum**	**Maximum**	**Mean**	**Std. Dev.**
Production (Q)	Tons (thousands)	3.01	221.14	68.30	45.00
Cotton Area (X_A_)	Hectares (thousands)	1.54	71.56	28.40	17.78
Labor (X_L_)	Workers (thousands)	25.91	1176.35	386.55	253.24
Capital (X_K_)	Hours (thousands)	100.14	5195.77	1778.59	1209.67
Materials (X_M_)	Tons (thousands)	0.34	15.67	6.22	3.89

Given that the quantities of the productive factors (labor, capital and materials) are estimated from the data on the cotton area, one possibility to verify that this procedure of estimation does not affect the final results significantly consists in using two alternative stochastic frontier production functions (instead of only one with four inputs). One of the stochastic frontier production functions includes only the input which is measured directly, X_A_:

$${\mathrm{Frontier A: Q}}_{it}=f(X_{Ait},t\text{;}\alpha )e^{v_{it}-u_{it}}$$ (9)

The other alternative stochastic frontier production function includes the three inputs which are estimated from X_A_:

$${\mathrm{Frontier B: Q}}_{it}=f(X_{Lit},X_{Kit},X_{Mit},t\text{;}\alpha )e^{v_{it}-u_{it}}$$ (10)

From the two alternative stochastic frontier production functions (9 and 10), we estimate four alternative models:

Model A1: It uses the frontier production function (A) and the ECM specification (Eq. 2).

Model A2: It uses the frontier production function (A) and the TEEM specification (Eq. 3).

Model B1: It uses the frontier production function (B) and the ECM specification (Eq. 2).

Model B2: It uses the frontier production function (B) and the TEEM specification (Eq. 3).

In order to estimate the four models and decompose TFP growth (Eq. 7) it is necessary to choose a functional form for the stochastic frontier production functions (Eq. 9 and Eq. 10). Taking into account the related literature (e. g., [13, 15, 16, 18]), and that the translog form is a flexible functional form, in this paper we propose a translog stochastic frontier production function:

$$\ln Q_{it}=\alpha_{0}+\sum_{j}\alpha_{j}\ln x_{ijt}+\alpha_{t}t+\frac{1}{2}\sum_{j}\sum_{l}\alpha_{jl}\ln x_{ijt}\ln x_{ilt}+\frac{1}{2}\alpha_{tt}t^{2}+\sum_{j}\alpha_{jt}(\ln x_{ijt})(t)+v_{it}-u_{it}$$ (11)

where *j* and *k* indicate the input quantities used in the production process.

Since in this study the statistical information on province-specific variables is not available, we create dummy variables (*D_i_*) for the different provinces to identify the possible heterogeneous behavior across them, with D_*i*_ being equal to 1 if the province is *i* and zero otherwise. Additionally, we incorporate a time variable to verify if inefficiency has increased or decreased in the analyzed period. Consequently, we specify the technical inefficiency effects (Eq. 3) as:

$$m_{it}=\delta_{0}+\sum_{i=2}^{N}\delta_{i}D_{it}+\beta t$$ (12)

From the estimation of the translog stochastic frontier production function (Eq. 11), the components of productivity change can be calculated. The technical efficiency levels (*TE*) and changes in technical efficiency (*CTE*), changes in allocative efficiency (*CAE*), technical progress (*TP*), and returns to scale (*RS*) are obtained as

$$TE=\frac{Q_{it}}{f(.)}=\exp(-u_{it})\;\;,\;CTE=\exp(-u_{it})-\exp(-u_{it-1})$$ (13)

$$CAE=\sum_{j}(\lambda_{j}-S_{j}){\dot{x}}_{j}$$ (14)

$$TP=\frac{\partial \ln Q_{it}}{\partial t}=\alpha_{t}+\alpha_{tt}t+\sum_{j}\alpha_{tj}\ln x_{ijt}$$ (15)

$$\begin{array}{l} RS=\sum_{j}\varepsilon_{j}=\sum_{j}\frac{\partial \ln Q_{it}}{\partial \ln x_{it}}=\;\sum_{j}[\alpha_{j}+\alpha_{jj}\ln x_{ijt}+\sum_{j\neq l}\alpha_{jl}\ln x_{ilt}+\alpha_{jt}(t)],\;\;[CSC=(\overset{}{RS}-1)\sum \lambda_{j}{\overset{\overset{}{}}{\dot{x}}}_{j}] \end{array}$$ (16)

## Results

The Maximum Likelihood estimates for the parameters of the four alternatives models can be obtained by using the FRONTIER 4.1 program [24], in which variance parameters are expressed in terms of $\sigma^{2}{}_{s}=\sigma^{2}{}_{u}+\sigma^{2}{}_{v}\;\;and\;\;\gamma =\frac{\sigma^{2}{}_{u}}{\sigma^{2}{}_{s}}$, where *γ* is an unknown parameter to be estimated. The adequacy of the models proposed and the different variants with regard to the type of distribution for inefficiency and the possible mu (μ) and eta (η) values have been tested. From the various alternatives, we selected (following the criterion of the likelihood ratio test) the results shown in table 2. The results of the four models are similar. Taking into account the values obtained for the log likelihood function, models A2 and B2 offer a better specification than models A1 and B1. On balance, model B2 presents the best specification.

**Table 2:** Maximum Likelihood estimates of the stochastic frontier production models.

	**Model A1**		**Model B1**		**Model A2**		**Model B2**	
**Variables**	**Coefficients**	**Standard error**	**Coefficients**	**Standard error**	**Coefficients**	**Standard error**	**Coefficients**	**Standard error**
Frontier Production Function								
Constant	0.249289	(0.114530)**	0.318254	(0.1267098)**	0.289005	(0.068000)***	0.339955	(0.074938)***
ln(XA)	0.563986	(0.072296)***			0.699767	(0.037490)***		
ln(XL)			-0.015121	(0.191079)			-0.035219	(0.160326)
ln(XK)			0.090003	(0.262512)			-0.267552	(0.228417)
ln(XM)			0.444902	(0.426944)			1.042037	(0.367086)***
T	-0.051286	(0.014198)***	-0.065920	(0.017786)***	-0.056645	(0.009873)***	-0.032985	(0.013500)**
½[ln(XA)]²	0.056628	(0.065249)			0.107206	(0.050583)**		
½[ln(XL)]²			-1.326062	(1.328960)			-2.713082	(1.109566)**
½[ln(XK)]²			-0.188999	(1.253958)			-1.498155	(1.117122)
½[ln(XM)]²			-2.452777	(2.239771)			-5.212243	(2.014632)***
½[t]²	0.009530	(0.002870)***	0.017803	(0.004239)***	0.010833	(0.002081)***	0.014033	(0.003458)***
ln(XL)ln(XK)			-0.058979	(0.609509)			-0.398795	(0.579186)
ln(XL)ln(XM)			1.348156	(1.448512)			3.092126	(1.210854)**
ln(XK)ln(XM)			0.683495	(1.567905)			2.067456	(1.380572)
ln(XA)(t)	0.062670	(0.008544)***			0.054666	(0.005415)***		
ln(XL)(t)			-0.118374	(0.059259)**			-0.157438	(0.056790)***
ln(XK)(t)			-0.025466	(0.076149)			-0.005993	(0.061207)
ln(XM)(t)			0.195844	(0.122160)			0.208574	(0.107433)*
Inefficiency Effects								
Constant					-0.370452	(0.375941)	-1.063039	(0.601952)*
D2					-0.298510	(0.342997)	-1.005989	(0.564093)*
D3					-0.386331	(0.365466)	-1.055583	(0.579813)*
D4					-0.285047	(0.350853)	-0.981394	(0.572738)*
D5					0.310457	(0.240579)	-0.147981	(0.362236)
D6					-0.338407	(0.298452)	-0.858379	(0.477231)*
D7					0.149254	(0.271432)	-0.339536	(0.399204)
D8					0.421313	(0.225923)*	0.005871	(0.332894)
D9					-0.077183	(0.321359)	-0.634279	(0.464208)
T					-0.120472	(0.026703)***	-0.154287	(0.045678)***
Sigma-squared	0.0705182	(0.009734)***	0.055808	(0.006899)***	0.154840	(0.040440)***	0.212099	(0.067683)***
Gamma	0.062285	(0.088807)	0.052890	(0.067910)	0.888835	(0.037669)***	0.926244	(0.029062)***
Mu	0.038352	(0.093079)	0.047633	(0.055249)				
Eta	0.134596	(0.030282)***	0.151787	(0.030046)***				
Log likelihood function	-23.082432		-6.236776		-0.725175		15.178791	
LR test of the one-sided error	24.227818		32.570919		68.942331		75.402053	
Total number of observations	171		171		171		171	

Note: ***, ** and * indicate significance at 1, 5 and 10% level, respectively

All variables appearing in natural logarithms were divided by their geometric mean prior to estimation. The time trend was at zero in 1999. As a result, the coefficients of the first-order terms of the variables in natural logarithms can be interpreted as production elasticities in that year evaluated at the geometric mean of the explanatory variables.

The first-order coefficients, *α_j_*, have the anticipated positive sign and they are statistically significant for the cotton area and materials inputs in models A1, A2 and B2, but they are not statistically significant for the capital and labor inputs in models B1 and B2. One possible explanation for the latter may be that these inputs are not used appropriately in the productive process.

The technical progress coefficient, *α_t_*, is statistically significant in the four models, but with negative sign; i. e., technical progress shifts the production frontier downward for a given level of inputs. This result probably suggests that there are other factors which are not considered in the production function and whose negative effects on output outweigh the positive effects of the possible technical progress.

The dummy variable coefficients of some provinces are statistically significant at the 10% level for model B2, which confirms that there are some province-specific effects. The negative and statistically significant coefficient for the time variable (*β*) in models A2 and B2 suggests that technical inefficiency in cotton production in Egypt tended to decrease during the studied period. As the eta (η) value is statistically different from zero (it takes a positive value in models A1 and B1), this implies that technical inefficiency in these provinces is not time-invariant; the level of technical efficiency improves in the analyzed period (as seen in table 3). The positive and statistically significant coefficient of η is compatible with the negative and statistically significant coefficient *β* (as we explained before). The variance parameter, gamma (*γ*), is statistically significant in models A2 and B2, and close to one, which suggests the relevance of technical inefficiency in explaining output variability. This estimate is consistent with the results of previous works (e.g. [25, 16]).

**Table 3:** Technical efficiency by year (total sample).

**Years**	**Model A1**	**Model B1**	**Model A2**	**Model B2**	**Minimum**	**Maximum**	**Mean**
1990	0.519	0.438	0.378	0.468	0.378	0.519	0.451
1991	0.557	0.482	0.420	0.565	0.420	0.565	0.506
1992	0.594	0.526	0.575	0.547	0.526	0.594	0.561
1993	0.629	0.569	0.710	0.745	0.569	0.745	0.663
1994	0.663	0.611	0.478	0.730	0.478	0.730	0.621
1995	0.695	0.650	0.491	0.517	0.491	0.695	0.588
1996	0.725	0.687	0.721	0.825	0.687	0.825	0.740
1997	0.752	0.722	0.767	0.808	0.722	0.808	0.762
1998	0.778	0.754	0.634	0.786	0.634	0.786	0.738
1999	0.802	0.783	0.749	0.853	0.749	0.853	0.797
2000	0.823	0.809	0.788	0.848	0.788	0.848	0.817
2001	0.843	0.832	0.801	0.830	0.801	0.843	0.827
2002	0.860	0.854	0.879	0.898	0.854	0.898	0.873
2003	0.876	0.872	0.903	0.909	0.872	0.909	0.890
2004	0.891	0.889	0.882	0.882	0.882	0.891	0.886
2005	0.903	0.903	0.905	0.899	0.899	0.905	0.903
2006	0.915	0.916	0.900	0.920	0.900	0.920	0.913
2007	0.925	0.927	0.879	0.901	0.879	0.927	0.908
2008	0.934	0.937	0.895	0.914	0.895	0.937	0.920
Mean(1990–2008)	0.773	0.745	0.724	0.781	0.724	0.781	0.756
Rate^a^	3.318	4.315	4.905	3.789	3.318	4.905	4.082

^(a)^Annual average percentage growth rate (1990–2008)

Table 3 shows the annual levels of the estimated technical efficiency, using the four proposed models in this study (for the total sample). By comparing the results obtained for the four models, it is possible to emphasize the following:

For the four models, the estimated annual levels of technical efficiency are similar. With respect to the average level of efficiency of the period 1990–2008, the estimates vary from a minimum level of 0.724 (model A2) to a maximum level of 0.781 (model B2), and the average of the four models is 0.756.

The four models consistently show that technical efficiency improves during the considered period. The average annual percentage growth rate is also similar for the four models, varying from a minimum rate of 3.318% (model A1) to a maximum rate of 4.905% (model A2), and with the average annual growth rate across the four models being 4.082%.

Results of TFP change decomposition for the four models are reported in Table 4. Changes for the total sample and the mean for every period are shown. From the results, we can highlight the following:

**Table 4:** TFP change decomposition^a^.

**Components**	**Model A1**	**Model B1**	**Model A2**	**Model B2**
TP	-0.0477	-0.0617	-0.0522	-0.0267
CSC	0.0346	0.0330	0.0248	0.0263
CAE	0.0000	-0.0114	0.0000	-0.0357
CTE	0.0230	0.0239	0.0287	0.0248
CTFP	0.0100	-0.0163	0.0013	-0.0113

^(a)^Mean changes for the total sample (1990–2008)

The four models offer similar results in the sense that the four models indicate the negative contribution of the technical progress component and the positive contributions of technical efficiency and scale components. In addition, models B1 and B2 coincide in the negative contribution of the allocative efficiency component.

The four models have similar results for the average magnitude of the contribution of the different components. The average negative contribution of TP varies from a minimum of -2.67% (model B2) to a maximum of -6.17% (model B1). The average positive contribution of CSC varies from a minimum of 2.48% (model A2) to a maximum of 3.46% (model A1). The average positive contribution of CTE is practically identical in the four models and around 2.5%. The average negative contribution of CAE is also similar for models B1 and B2, and it is higher for the model that considered the specific effects of inefficiency (-3.57% for model B2 compared to -1.14% for model B1).

The main difference in the results is due to the fact that models A1 and A2 are more restricted. These models only include one productive factor and therefore they do not allow to quantify the behavior of allocative efficiency. In these models the allocative efficiency is assumed not to change, and in this case a slight average productivity growth is estimated (1% for model A1 and 0.13% for model A2). On the other hand, taking into account the possible changes in allocative efficiency (models B1 and B2) the estimated average change in TFP is negative (-1.63% for model B1 and -1.13% for model B2).

The negative contribution of the technical progress component must be considered in light of its definition as a residual. A negative value of this component must be interpreted as a downward movement of the production frontier. But this does not mean that in average there was no technical progress in cotton production; what this result probably indicates is that its contribution was small, and that there are possibly other factors (which have not been explicitly considered in the model) whose negative effect on production outweighs the small positive effect of the possible technical advances (e.g. land quality, climate changes, plant diseases, plant viruses, etc).

The positive value for the changes in the scale component means that the cotton production sector took advantage of the economies of scale. With the statistical information available it is not possible to identify the specific factors responsible for this improvement in scale economies.

The changes in allocative efficiency exerted a negative effect on TFP growth (models B1 and B2). The presence of allocative inefficiency shows that, during the period of analysis, input prices were not equal to the value of their marginal product and thus these inputs were not allocated in the correct proportions; i.e., the input combination that minimizes the cost of production was not chosen.

The positive value for the changes in technical efficiency shows that the gap between the production frontier and the actual cotton production was squeezed throughout the analyzed period.

Finally, if we consider the more complete models (B1 and B2), it is possible to conclude that the progress in technical efficiency and economies of scale does not compensate for the negative effects on productivity caused by the misallocation of the productive factors (that is, the presence of allocative inefficiency) and for the negative effects of the other factors included in the technical progress component.

Table 5 shows the evolution of productivity and its components over time. We take as a reference model B2, which is the model that offers the best specification and also allows to analyze the behavior of allocative efficiency. From the analysis of this evolution, some interesting considerations can be drawn:

**Table 5:** TFP change decomposition by years (model B2)^a^.

**Years**	**TP**	**CSC**	**CAE**	**CTE**	**CTFP**
1991	-0.1042	0.0338	-0.1270	0.0974	-0.1000
1992	-0.1387	0.0676	-0.2629	-0.0180	-0.3520
1993	-0.1045	0.0010	-0.0243	0.1977	0.0699
1994	-0.0424	-0.0433	0.3500	-0.0149	0.2494
1995	-0.0566	-0.0061	-0.3830	-0.2137	-0.6593
1996	-0.0237	0.0269	-0.0591	0.3083	0.2524
1997	-0.0698	0.0117	-0.1928	-0.0168	-0.2677
1998	0.0271	0.0098	0.2508	-0.0216	0.2661
1999	-0.0684	0.1160	-0.3081	0.0667	-0.1938
2000	-0.0814	-0.0104	0.2772	-0.0054	0.1800
2001	-0.0379	-0.0753	-0.0953	-0.0176	-0.2260
2002	0.0910	0.0214	0.0717	0.0680	0.2521
2003	0.0315	0.1027	-0.2203	0.0110	-0.0751
2004	0.0199	-0.0861	0.0816	-0.0274	-0.0119
2005	0.0341	0.1204	-0.0900	0.0168	0.0813
2006	0.0256	-0.0608	0.0593	0.0215	0.0455
2007	0.0128	0.2256	-0.0689	-0.0189	0.1506
2008	0.0054	0.0192	0.0978	0.0129	0.1353
Mean (1991–2008)	-0.0267	0.0263	-0.0357	0.0248	-0.0113

^(a)^Changes for the total sample

Although during the analyzed period the changes in TFP and its components are not high in average, the annual variations are much higher. This is largely due to the fact that the variability of the output and the inputs is also high (Table 1).

The evolution of the changes in productivity and in its components does not show a clear trend, but in the last years a certain stabilization and a slight progress in the evolution of TFP can be observed.

TP is the component that experienced the least variability. This component presents a clear change of trend from the year 2002, when it started to contribute positively to the growth of productivity.

CTFP is the indicator that offers the highest annual variability, followed by the allocative efficiency component. The latter is also the component that has the greatest relative importance in determining the behavior of TFP.

Results of TFP growth decomposition by provinces are reported in Table 6. According to the information reported in Table 4, the results for model B2 show that the mean of CTFP of the total sample for the period 1990–2008 is -1.13% per year. The unfavorable evolution of productivity is the result of negative average contributions of TP (-2.67%) and CAE (-3.57%), which are not offset by the positive average contributions of CSC (2.63%) and CTE (2.48%). But the changes in productivity and in its components are not homogeneous across provinces. In three provinces (Dakahlia, Behairah and Menia) the average changes in productivity are positive. The components CSC, CAE and CTE maintain the sign of their contribution across provinces, although with varying magnitude. The TP component has a positive contribution in two provinces (Kafr Elshikh and Behairah) but also with different size in each of them.

**Table 6:** Results of TFP change decomposition by provinces (model B2)^a^.

**Provinces**	**TP**	**CSC**	**CAE**	**CTE**	**CTFP**
Dakahlia	-0.0054	0.0148	-0.0216	0.0151	0.0028
Sharkia	-0.0214	0.0230	-0.0329	0.0234	-0.0079
Kafr Elshikh	0.0058	0.0056	-0.0310	0.0195	-0.0002
Gharbia	-0.0297	0.0286	-0.0402	0.0250	-0.0163
Menoufia	-0.0649	0.0540	-0.0523	0.0302	-0.0331
Behairah	0.0164	0.0085	-0.0249	0.0086	0.0085
Beni Suef	-0.0436	0.0310	-0.0443	0.0337	-0.0231
Fayoum	-0.0536	0.0191	-0.0360	0.0366	-0.0340
Menia	-0.0437	0.0527	-0.0384	0.0311	0.0017
Total simple	-0.0267	0.0263	-0.0357	0.0248	-0.0113

^(a)^Mean changes for the period 1990–2008

## Conclusions and Policy Implications

Considering a situation of scarce statistical information, the focus of the study is novel in the sense that it proposes four alternative empirical models, with the purpose of looking for convergence in the results to validate to some extent the conclusions and to minimize the effect of working with a deficient database.

Indeed, the results of the estimation for the four models are consistent. The four models provide similar results for the technical efficiency (considering the total sample), and they all indicate the negative contribution of the technical progress component and the positive contributions of technical efficiency and scale components to total productivity growth. In addition, models B1 and B2 coincide in the negative contribution of the allocative efficiency component.

These results provide evidence that the cotton production system in Egypt offers some deficiencies. Although the levels of technical efficiency improve during the considered period, in this production system the total productivity does not increase. The results for model B2 show that the mean of CTFP of the total sample for the period 1990–2008 is -1.13% per year. The unfavorable evolution of productivity is mainly due to the negative average contributions of CAE (-3.57%) and TP (-2.67%). Therefore, this lack of productivity growth can be one of the causes of cotton production decline.

The allocative efficiency component is the one with the greatest relative importance in determining the negative behavior of TFP. The presence of allocative inefficiency provides evidence that, during the period of analysis, the inputs were not allocated in the correct proportions; i.e., the input combination that minimizes the cost of production was not chosen. This result might be related with the characteristics of the exploitations and the workers. If the farms are small (and in fact the average farm size in Egypt is about 0.6 hectare, which hinders the use of machinery) and the workers are inadequately trained, this might prevent the inputs from being used adequately and in the correct proportions [26]. And the inputs not being used appropriately might be one of the causes for them not appearing significant in the productive system; as a matter of fact, in this study the capital and labor inputs do not show to be significant in the stochastic frontier production models.

The negative contribution of technical progress does not mean that in average there was no technical progress in cotton production; what this result probably indicates is that its contribution was small, and that there are possibly other factors whose negative effect on production outweighs the small positive effect of the possible technical progress. Another possible reason is that the existent technology might not be used appropriately.

This production system leads to varying results in the different cotton-producing provinces of Egypt, since the results for the changes in productivity and in its components are not homogeneous across them. This might suggest that some provinces have worse conditions for the production of cotton.

In light of these research results, and from the point of view of establishing an agricultural policy for the cotton production system in Egypt, some recommendations can be made:

Policy makers should improve the database of the cotton production sector. Only with a good database it is possible to get to know the productive reality of the cotton production sector appropriately, and a sufficient knowledge of this productive reality is required in order to establish an effective agricultural policy. This improved database should contain sufficient statistical information at the disaggregated levels (at provincial and farm levels). With this kind of statistical information it would be possible to identify the specific factors that influence allocative efficiency and technical progress.

Policy makers should aim to improve the total productivity of cotton with the purpose of reducing the production costs and increasing the degree of competitiveness of the Egyptian cotton production. It would therefore be useful to identify and to tackle the factors that cause the negative contributions of the allocative efficiency and the technical progress components to total productivity growth. Although these factors are not precisely identified due to the lack of statistical information, following previous literature in the agricultural field (e. g., [27, 17]) some effective policy measures (such as providing better extension services and farmer training programs) can be proposed to improve the capacity of farmers. If the farmers have greater capacity, they are able to allocate their resources more efficiently and make a better use of the available technology.

Finally, policy makers should take into account that the behavior of productivity and its components is not homogeneous across provinces. Therefore, it seems reasonable to propose specific measures for each of them and to analyze the possibility of orientating production towards the provinces where productivity shows a relatively better behavior.
